# Antimicrobial Profiling of Bacteria Isolated from Fish Sold at Informal Market in Mufakose, Zimbabwe

**DOI:** 10.1155/2019/8759636

**Published:** 2019-05-02

**Authors:** Claudious Gufe, Tinashe Canaan Hodobo, Bernard Mbonjani, Otlia Majonga, Jerikias Marumure, Shuvai Musari, Gilbert Jongi, Pious Vengesayi Makaya, Jairus Machakwa

**Affiliations:** ^1^Division of Veterinary Services, Diagnostics and Research Branch, Central Veterinary Laboratories, P.O. Box CY 551, Causeway, Harare, Zimbabwe; ^2^Department of Biological Sciences, Faculty of Science, Bindura University of Science Education, P. Bag 1020, Bindura, Zimbabwe; ^3^Division of Veterinary Services, Veterinary Public Health Branch, P.O. Box CY551, Causeway, Harare, Zimbabwe

## Abstract

The number of infections caused by antibiotic resistant bacteria is rising worldwide. Fish from multisource pollution waters can harbour multidrug-resistant bacteria that can be disseminated to humans through eating or contact of contaminated fish. A cross-sectional study was carried out to (i) isolate and phenotypically identify bacteria from 36 fish samples from informal market in Mufakose, Harare, and (ii) determine the antibiotic sensitivity pattern of the isolated bacteria against ten available antibiotics (ampicillin 10 *μ*g, gentamycin 30 *μ*g, penicillin G 10 *μ*g, erythromycin 15 *μ*g, tetracycline 30 *μ*g, kanamycin 30 *μ*g, neomycin 10 *μ*g, cloxacillin 5 *μ*g, lincomycin 15 *μ*g, and sulfamethoxazole 25 *μ*g) using the Kirby–Bauer disk agar diffusion method. Eight bacterial genera were isolated and identified, and they were *Escherichia*, *Aeromonas*, *Staphylococcus*, *Pseudomonas*, *Citrobacter*, *Klebsiella*, *Enterobacter*, and *Proteus*. Among the isolates, *Escherichia coli* was isolated most frequently (44%) followed by *Staphylococcus aureus* (19%), *Enterobacter aerogenes* (7%), *Aeromonas* spp. (5%), *Proteus mirabilis* (5%), *Citrobacter* (5%), and coagulase-negative *Staphylococci* (5%) and the least frequent were *Klebsiella* (3%) and *Pseudomonas* (3%). All isolates were susceptible to gentamycin. Varying antibiotic resistance rates were observed to lincomycin (100%), ampicillin (81%), penicillin (67%), erythromycin (65%), tetracycline (63%), neomycin (61%), cloxacillin (43%), kanamycin (24%), and sulphamethoxazole (13%). All the isolates were multidrug-resistant (resistant to at 3 or more drugs tested) except *Proteus mirabilis*. *Proteus mirabilis* has multiple antibiotic resistance (MAR) index of 0.2, and the other isolated bacteria had MAR indexes greater than 0.2 ranging from 0.3 to 0.7. Those MAR indexes above 0.2 showed that the bacteria isolates are from a high risk source where antibiotics were frequently used, possibly from sewage effluents. Isolation of enteric bacteria such as *Escherichia coli* is an indication of faecal contamination, and this poses a high risk to animal and human health. These significant findings call for effective risk assessment models and management plans that protect human, animal, and environmental health.

## 1. Introduction

In many countries, fish are consumed and are considered to be a good source of dietary protein. The fish provide high-quality essential proteins. According to FAO [1], fish is the most important single source of high-quality protein providing about 16% of the animal protein consumed by the world's population. In Africa, fish constitutes about 17% of animal protein consumed [2]. Fish has high nutritional values such as low saturated fat and good source of essential fatty acids, the omega-3 fatty acids which cannot be synthesized by the human body. Fish are known to contain low fat and low cholesterol and to be highly digestible making them suitable to the infants, children, and elderly. It is relatively cheap compared to other meat products such as beef and poultry; hence, it is more affordable to most people.

From the perspective of microbiology, fish and related products are a potential health risk since they harbour important human pathogenic bacteria on or inside them. Bacterial infections might arise through improper handling and through the consumption of badly prepared fish. Several bacterial genera such as *Escherichia*, *Listeria*, *Pseudomonas*, *Klebsiella*, and *Salmonella* were isolated from fish and can indicate multisource pollution [3, 4]. Most people get infected through contact while handling water or other constituents of fish living environments. The development of infectious diseases is also markedly affected by the physiological status of the consumer, for instance, immunocompromised or stressed individuals are quite susceptible to opportunistic infection as exemplified by people living with HIV and AIDS [5].

Banquero et al. [6] described aquatic systems as genetic reactors or hotspots for AMR genes where significant genetic exchange and recombination can occur, which can shape the evolution of future resistance profiles. Bacteria acquire resistance either through mutations in their genetic material or through a process called horizontal gene transfer. Resistance to antibiotics can be conferred by chromosomal or mobile genetic elements and achieved using four main strategies, namely, reduction of membrane permeability to antibiotics, drug inactivation, and rapid efflux of the antibiotics and mutation of cellular target [7, 8]. A large part of the exchange of resistance genes takes place in the gastrointestinal tract [8]. After excretion, the potential resistant bacteria end up in the environment and near food products of animal origin. These food products are an important source of human infection, thus causing an increase in use of antibiotics, which favours the emergence of antibacterial resistance [9].

The number of infections caused by antibiotic-resistant bacteria is rising worldwide [8]. Some bacterial infections, e.g., salmonellosis, do not require treatment; however, if the infection is serious or invasive, antimicrobial drugs are commonly administered [7, 8]. Moreover, the overuse of antimicrobial drugs to prevent or treat infections in human and veterinary medicine contributes to the increased frequency and dissemination of AMR [10]. Irrational use of antimicrobials is associated with increased selection pressure on bacterial populations and favours survival and multiplication of resistant bacteria. Antimicrobial resistance is a naturally occurring phenomenon, and the resistance rates are kept at reasonably low levels; however, increased AMU can drive the rise in the rate of resistance. Drug resistance in isolates originating from wildlife, which are not influenced by selective pressures of antimicrobial drugs, is thought to be low. Nevertheless, Levy and Marshall [8] proposed that the fastest way to get rid of resistant bacteria is to outnumber them with sensitive bacteria.

Fish usually succumb to opportunistic bacterial infection due to physiological imbalance. Stress factors such as nutritional deficiencies, poor quality water, and overstocking are predisposing factors in the development of fish infections. Pathogenic bacteria are classified as indigenous or nonindigenous. Pathogenic bacteria common in fish include *Aeromonas* species, which is ubiquitous in water in freshwater environments [11]. The same authors also added that indigenous bacteria which include *Aeromonas*, *Clostridium*, and *Vibrio* are widely distributed in aquatic environments in the warm tropical zones and estuarine environment. *Salmonella*, *E. coli*, and *Shigella* bacteria survival in water depends on many parameters such as biological (interactions with other bacteria) and physical factors such as temperature. The occurrence of nonindigenous bacteria such as *E. coli*, *Salmonella*, and *Shigella dysenteriae* in fish is normally as a result of faecal contamination.

Consequently, it has been strongly recommended that programmes to monitor the usage of antimicrobial agents and occurrence of antimicrobial resistance among animals, plants, and humans should be established [12]. This has been initiated in some countries; however, a major obstacle has been the lack of coordination and standardization both, over time or between countries, complicating comparisons. The appearance of antibiotic resistance among bacteria from animals has raised considerable concern due to the potential for transfer of resistant pathogens and commensal bacteria to the human population [13]. The safety of eating fish from informal market is not known; therefore, there is a need to assess and investigate the potential risk posed to human health by consuming fish sourced from the informal market. The fish on the informal market are from heavily contaminated Lake Chivero with untreated sewage and agricultural and industrial wastes. The main objective of this study was to isolate and identify bacteria from fish at the informal market in Mufakose and to determine the level of antibiotic resistance rates of the isolated bacteria against ten antibiotics.

## 2. Materials and Methods

### 2.1. Study Design and Sample Collection

This was a cross sectional study in which a total of 36 fish samples were collected from vendors along shopping centres in Mufakose, Harare, Zimbabwe, from mid-July to August 2017. A total of 36 fish samples were collected. Twelve fish samples were collected at the first visit and was repeated three times. Fish bought from vendors in Mufakose were collected in sterile plastic bags labelled with identities derived from location of collection. Fish samples were transported to the laboratory for testing in cooler boxes and tested on the same day. Samples were collected from the vendors using random sampling. Mufakose is a high-density suburb with a lot of informal markets along streets or and shopping centres.

### 2.2. Isolation and Identification of Bacteria

Ten grams of fish were cut into small pieces, placed in a labelled bottle containing peptone water, listeria-selective broth, and buffered peptone water. The contents were homogenised. The homogenate from peptone water was streaked using a sterile loop on blood agar and MacConkey, Skirrows, and *Campylobacter* agars for *Vibrio* and *Campylobacter* [14]. The streaked plates were incubated some aerobically and anaerobically at 37°C for 24 hours. For *Campylobacter*, incubation was done in anaerobic jars provided with *Campylobacter* gas generating sachets (Oxoid) [14]. Homogenate in *Listeria* selective broth was incubated for 24 hours at 37°C. After incubation, the broth was streaked on *Listeria* agar and incubated for 24 hours at 37°C [14]. Homogenate in buffered peptone water was incubated for 24 hours at 37°C. After incubation, 1 ml of the homogenate was transferred into Rappaport Vassiliadis (RV) and incubated for 24 hours at 37°C. On day three, a loopful of the sample was streaked on Xylose lysine deoxychocolate (XLD) and incubated for 24 hours at 37°C [14].

### 2.3. Morphology and Biochemical Characterization of Isolated Bacteria

**Figure 1:**
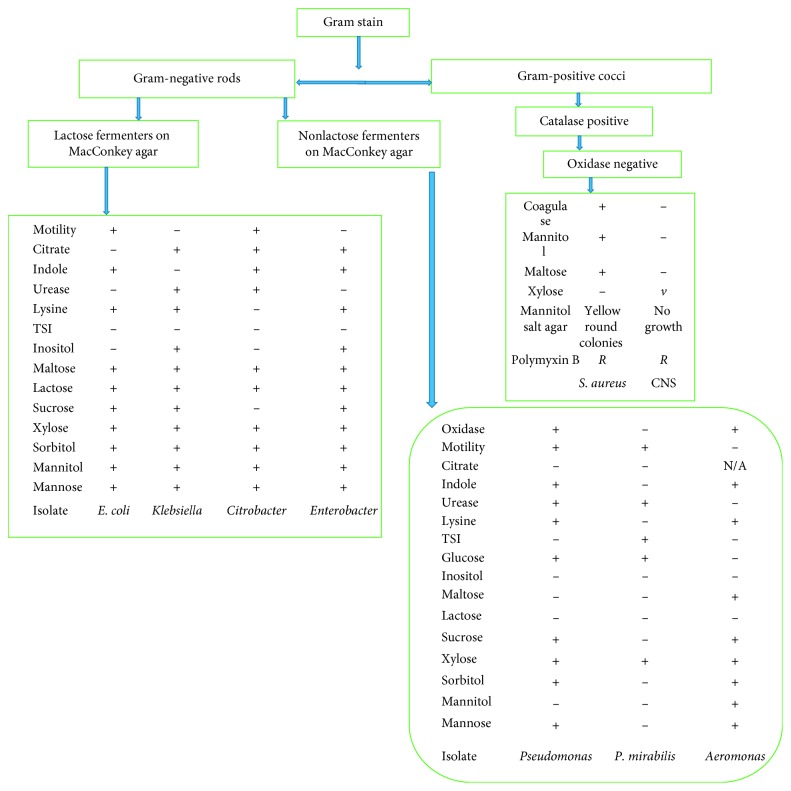
Flow diagram showing Gram staining and some biochemical tests used to identify different bacterial isolates.

The bacteria were identified using morphological characteristics, Gram staining, and biochemical tests as indicated in Figure 1 with some subcultured on selective differential media [14]. To identify the bacteria, morphological characteristics, a range of biochemical tests such as oxidase, motility, indole, citrate, lysine decarboxylase, urease, and triple sugar iron (TSI), and a range of sugars were performed. Autoclaved fish and uninoculated media were used as a negative controls and during biochemical testing, different ATCC organisms from Microbiologics (*Staphylococcus aureus* ATCC 33862, *Escherichia coli* ATCC 25922, *Klebsiella pneumoniae* ATCC 13883, *Citrobacter freundii* ATCC 8090, and *Pseudomonas aeruginosa* ATCC 27853) were used as positive controls.  Table 1 shows some of the morphological characteristics used for bacterial identification such as colony colour, smell, and haemolysis on sheep blood agar. MacConkey agar was used as a selective and differential media, differentiating lactose fermenters from nonlactose fermenters.

### 2.4. Antibiotic Profiling

At least 4-5 well isolated colonies of the same morphological type from a pure culture were selected and swabbed using a sterile cotton swab [14]. The colonies were transferred into sterile 0.85% physiological saline water (PBS), and the bacteria emulsified until the turbidity is similar to the 0.5 McFarland standard. Another sterile swab was immersed in PBS suspension, and the swab was pressed against the walls of the bijoux bottle above the fluid level to remove excess fluid. The swab was then streaked in 3 different directions over the surface of a plate of Mueller–Hinton agar (MHA) such that a uniform well-spread-out inoculation is achieved. The inoculated plateswere allowed to stand for 3–5 minutes so that the inoculum can dry. The antibiotic disc dispenser was retrieved from the refrigerator and left on the bench for 15 minutes at room temperature. The standard antibiotic disks (Oxoid) containing sulfamethoxazole-trimethoprim combination (25 mcg), penicillin (10 mcg), tetracycline (30 mcg), gentamycin (10 mcg), erythromycin (15 mcg), ampicillin (10 mcg), kanamycin (30 mcg), neomycin (10 mcg), cloxacillin (5 mcg), and lincomycin (15 mcg) were dispensed onto well-labelled inoculated MHA plates using the disc dispenser. The antibiotic disks used were based on their availability at the laboratory at the time of the study. The plates were allowed to stand for few minutes and were incubated at 37°C for 24 hours within 15 minutes of applying [14]. Antibiotic sensitivity was checked by measuring the zone of inhibition (zone of clearance) from the back of the plate to the nearest mm using a ruler or caliper. The zone of inhibitions were recorded and used to establish if the bacterial isolates were resistant, intermediate, and susceptible using reference books and WHONET. The bacteria were reported as sensitive (S), intermediate (I), or resistant (R) to each of the antibiotics used in the test.

### 2.5. Multiple Antibiotic Resistance (MAR) among the Isolated Bacteria

The MAR index when applied to a single isolate is defined as **a/b**, where “**a**” represents the number of antibiotics to which the isolate was resistant and “**b**” represents the number of antibiotics to which the isolate was exposed. For example, if the isolate was exposed to twelve antibiotics and was tolerant to six antibiotics, the index for the isolate would be 6/12 or 0.50 [15]. The MAR index was calculated for all the bacterial isolates.

### 2.6. Data Analysis

Prevalence rates of bacteria isolates was calculated as the number of times the bacteria species was identified over the total number of all the bacteria species identified. Resistance rates were calculated for each antibiotic and each bacterial isolates as the number of species isolates resistant over the total number of species tested. The overall resistance rates of each antibiotic were calculated as the number of bacteria resistant to antibiotic over total number of bacteria isolates tested.

## 3. Results

### 3.1. Bacteria Isolation and Identification

**Figure 2:**
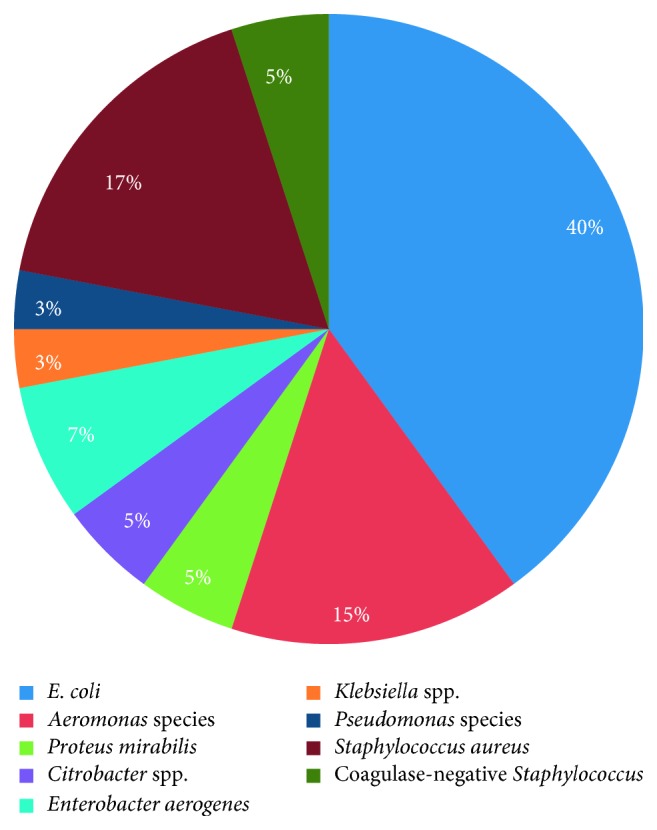
Prevalence rate of the isolated bacteria.

The bacteria isolated were *E. coli*, *Aeromonas* species, *Citrobacter* species, *Staphylococcus aureus*, *Klebsiella* species, *Enterobacter aerogenes*, *Proteus mirabilis*, *Pseudomonas* species, and coagulate-negative *Staphylococci* which were identified using morphological properties, Gram staining, and series of biochemical tests. *Escherichia coli* (24 out of 60) was the most dominant species among the isolated isolates from fish samples as indicated in Figure 2. Figure 2 below shows the percentage prevalence of the isolated bacteria: *E. coli* (40%), *Staphylococcus aureus* (17%), *Aeromonas* species (15%), *Enterobacter aerogenes* (7%), *Proteus mirabilis* (5%), *Citrobacter* species (5%), coagulase-negative *Staphylococci* (5%), *Pseudomonas* (3%), and *Klebsiella* (3%).

### 3.2. Antimicrobial Susceptibility and Resistance Testing

All the identified isolates were susceptible to gentamycin, and all the isolates were resistant to lincomycin. The isolated bacteria showed variable resistance rates to each antibiotic as shown in Table 2. Overall resistance rates for each antibiotic for all the organisms identified are lincomycin (100%), ampicillin (81%), penicillin (67%), erythromycin (65%), tetracycline (63%), neomycin (61%), cloxaxillin (43%), kanamycin (24%), and sulphamethaxole (13%). All the isolates have a 100% resistance to at least three antibiotics used except for *Proteus mirabilis* which was 100% resistant to only two antibiotics. We concluded that all the bacterial isolates from fish sold at informal market were multidrug resistant except for *Proteus mirabilis*. Few species of *Citrobacter* and *Klebsiella* were intermediate and was not considered resistant but was bunched in the susceptible group.

The MAR index of all isolates show values higher than 0.2 except *Proteus mirabilis* which has MAR index of 0.2 (Table 3). From Table 3, all the isolated bacteria were multidrug resistant (resistant to 3 or more antibiotics) except for *Proteus mirabilis*. Table 3 also shows the MAR indexes of the isolated bacteria ranging from 0.2 to 0.7. *Escherichia coli*, *Enterobacter aerogenes*, and *Staphylococcus aureus* have the highest MAR index value of 0.7. The trend of antimicrobial resistance is shocking for the bacterial species isolated from fish sold at informal market.

## 4. Discussion

### 4.1. Bacteria Isolation and Identification

Most outbreaks of food poisoning associated with fish are derived from the consumption of raw or insufficiently heat-treated fish, which may be contaminated with bacteria from water environment (*Vibrio* spp. and *C. botulinum*) or terrestrial sources (*C. perfringens*, *Salmonella* spp., *Shigella* spp., *Staphylococcus* spp., and *V. cholerae*), or fish products recontaminated after heat processing. In the case of poor hygiene, the contamination of fish and fish products may increase due to unsanitary procedures, the rotation of the assigned duties of workers, handling, and airborne microorganisms during packing of the product. The 8 bacteria genera isolated in this study (Figure 1) corroborate to 4 bacteria genera isolated from edible fish in Zimbabwe in [3] (*Pseudomonas*, *Escherichia*, *Klebsiella*, and *Proteus*) and to 3 bacteria genera in [4] (*Escherichia*, *Staphylococcus* and *Pseudomonas*). This study also corroborates internationally to 3 genera (*Pseudomonas*, *Escherichia*, and *Staphylococcus*) isolated from fish in India in [16]. The prevalence rates of the isolated bacteria shown in Table 1 (*E. coli* was the most abundant followed by *S. aureus*) were not in agreement with those reported by [4] (*S. aureus* was the most abundant followed by *E. coli*). Isolation and identification of enteric bacteria (*E. coli*, *P. mirabilis*, *Klebsiella*, *Citrobacter* species, and *E. aerogenes*), *S. aureus*, CNS, and *Pseudomonas* species indicate multisource pollution of fish from sewage effluents, humans during handling, and industrial and agricultural wastes. From this study, it is found that the presence of haemolytic *E. coli* can cause diarrhoea mainly in kids and immunosuppressed individuals. The presence of *E. coli* and other coliforms (*Klebsiella*, *Citrobacter* species, and *E. aerogenes*) in food is a clear indication of environment and faecal pollution either from humans and or from animals as well as poor handling practices [17–19]. The presence of *E. coli* can indicate the presence of other disease-causing pathogens (health risk) in the fish even if they were not isolated. According to [4], enteric bacteria were isolated from fish ponds, where animal manure was added indicating faecal contamination. In Harare, there are challenges in sewage management, and raw sewage sometimes ends up in fresh water sources like rivers. When sewage pipes burst, raw sewage contaminates the environment including water sources. Some sewage contaminated river systems flow into Lake Chivero where most of the fishing activities are done. There is also a problem of bursting of sewage pipes, thus exuding raw untreated sewage from the high-density suburbs. Fish are contaminated from the sewage and industrial wastes [20] as well as from poor handling techniques by vendors.

*Proteus mirabilis* is also a commensal in warm bodied animals; therefore, its present might be due to faecal contamination. *P. mirabilis* is an opportunistic pathogen which primarily affects an individual whose immune system is compromised. The presence of *Aeromonas* spp. indicates that the fish were infected in the lake. This is in concurrence with [14], stipulating that pathogenic bacteria common in fish include *Aeromonas* species which is ubiquitous in water in freshwater environments. The presence of *S. aureus* and CNS means that the fish were contaminated from human and animal wastes at the source (lake) or during handling. In this study, there was no *Salmonella*, *Listeria*, *Vibrio*, and *Shigella* genera, which were isolated by [3, 4, 16, 20]. In our study, we isolated more bacteria genera (8) than that in [3, 4, 16, 20].

### 4.2. Antibiotic Resistance

The study of antibiotic resistance in pathogenic bacteria from fish is important, as it might indicate the extent of alteration of water ecosystems by anthropogenic activities. Actually, water bacteria could be indigenous to aquatic environments, or exogenous, transiently and occasionally present in the water as a result of shedding from animal, vegetal, or soil surfaces. The resistance of the strains to antibiotics could be explained by the possibility of the heavy use of these compounds in aquaculture, several of which are non-biodegradable, thus increasing antibiotic selective pressure in water, facilitating the transfer of antibiotic-resistant determinants between aquatic bacteria, including fish and human pathogens, and allowing the presence of residual antibiotics in commercialized fish and shellfish products [12, 21, 22].

The identified isolates were multiresistant to various antibiotics used except for *Proteus mirabilis*. Indeed, all bacteria were sensitive to gentamicin, and this corroborates a study by [3]. Baquero et al. [6] found that all strains of *A. hydrophila* were sensitive to gentamicin, and this is in agreement with all the *Aeromonas* species tested in this study. Antimicrobials in waste water are increasingly found and potentially have an important role in the rise and selection of antimicrobial resistance in the environment [12, 21]. The MAR index of all isolates show values higher than 0.2 except for *Proteus mirabilis* which has a MAR index of 0.2. Those multidrug-resistant bacteria with MAR indexes greater than 0.2 indicate that they are from the high-risk source where antibiotics are frequently used, possibly faecal contamination. Due to indiscriminate use of antibiotics, the microorganisms might have developed resistance towards several antibiotics.

## 5. Conclusion

Raw sewage poured into lakes and rivers contaminates these water bodies with bacteria originating from human waste. Fish harvested from contaminated water bodies meant for human consumption are potential sources of human pathogenic bacteria. The contaminated water can also be associated with illness in the fish. The isolated multidrug-resistant bacteria pose high risk to human, animal, and environment health. Strict rules and monitoring activities combined with food safety training fishermen, vendors, and consumers on various aspects of good hygiene practices are strongly recommended.

## Figures and Tables

**Table 1:** Colony morphology of the suspected bacteria.

Blood agar	MacConkey agar	Suspected bacteria
Yellow round raised smooth shiny colonies	Tiny pink colonies	*Staphylococcus* species
Grey round flat haemolytic with coliform smell	Bright pink round colonies	*E. coli*
Grey round raised with coliform smell	Pink round colonies	Coliforms
Grey, flat, round, haemolytic colonies with foul smell	Pale round raised colonies	*Aeromonas* species
Blue-green flat, round, haemolytic fruity smell	Pale round raised colonies	*Pseudomonas* species
Large grey mucoid colonies	Pale-pink colonies	*Klebsiella*/*Enterobacter* species
Large grey mucoid colonies	Pink round colonies	*Klebsiella*/*Enterobacter* species
Grey swarming colonies with foul smell	Pale round colonies	*Proteus* species

**Table 2:** Resistance rate of bacteria to antibiotics.

Bacteria	PEN	SXT	ERY	GEN	NEO	KAN	CLO	AMP	TET	LIN
*E. coli*	100	0	50	0	100	0	50	88	67	100
*Aeromonas* species	0	100	100	0	0	100	0	0	0	100
*Proteus mirabilis*	0	0	0	0	0	0	0	0	100	100
*Citrobacter* spp.	67	0	100	0	100	0	0	100	67	100
*Enterobacter aerogenes*	100	25	100	0	100	0	100	100	100	100
*Klebsiella* spp.	100	0	50	0	100	0	50	50	50	100
*Pseudomonas* species	0	0	100	0	0	0	100	100	0	100
*Staphylococcus aureus*	40	30	70	0	0	100	40	100	80	100
Coagulate-negative *Staphylococcus*	0	0	100	0	0	0	0	100	0	100

**Table 3:** Multidrug resistance and MAR index of the isolated bacteria.

Isolates	MDR isolate	MAR index
*Citrobacter* species	Positive	0.5
*E. coli*	Positive	0.7
*Enterobacter aerogenes*	Positive	0.7
*Klebsiella* species	Positive	0.5
*Proteus mirabilis*	Negative	0.2
*S. aureus*	Positive	0.7
CNS	Positive	0.3
*Pseudomonas aeruginosa*	Positive	0.4
*Aeromonas* species	Positive	0.4

## Data Availability

The data used to support the findings of this study are available from the corresponding author upon request.

## Additional Points

*Recommendation.* (i) Thorough investigation of both phenotypic and genotypic antibiotic resistance on pathogenic bacteria isolated from fish and water bodies in Zimbabwe must be done. (ii) Also, people must thoroughly cook the fish they buy from open market to prevent infections from these pathogens. (iii) The city must minimize or control sewage effluent to enter the water bodies to prevent pollution.
